# Adolescent Premature Ovarian Insufficiency Following Human Papillomavirus Vaccination: A Case Series Seen in General Practice

**DOI:** 10.1177/2324709614556129

**Published:** 2014-10-28

**Authors:** Deirdre Therese Little, Harvey Rodrick Grenville Ward

**Affiliations:** 1 Bellingen District Hospital, Bellingen, New South Wales, Australia; 2 University of New South Wales, Coffs Harbour, New South Wales, Australia

**Keywords:** premature ovarian failure, amenorrhea, human papillomavirus vaccination, ovarian insufficiency, menopause

## Abstract

Three young women who developed premature ovarian insufficiency following quadrivalent human papillomavirus (HPV) vaccination presented to a general practitioner in rural New South Wales, Australia. The unrelated girls were aged 16, 16, and 18 years at diagnosis. Each had received HPV vaccinations prior to the onset of ovarian decline. Vaccinations had been administered in different regions of the state of New South Wales and the 3 girls lived in different towns in that state. Each had been prescribed the oral contraceptive pill to treat menstrual cycle abnormalities prior to investigation and diagnosis. Vaccine research does not present an ovary histology report of tested rats but does present a testicular histology report. Enduring ovarian capacity and duration of function following vaccination is unresearched in preclinical studies, clinical and postlicensure studies. Postmarketing surveillance does not accurately represent diagnoses in adverse event notifications and can neither represent unnotified cases nor compare incident statistics with vaccine course administration rates. The potential significance of a case series of adolescents with idiopathic premature ovarian insufficiency following HPV vaccination presenting to a general practice warrants further research. Preservation of reproductive health is a primary concern in the recipient target group. Since this group includes all prepubertal and pubertal young women, demonstration of ongoing, uncompromised safety for the ovary is urgently required. This matter needs to be resolved for the purposes of population health and public vaccine confidence.

## Introduction

Premature ovarian insufficiency (POI) has been defined as hypergonadotropic hypogondism developing before age 40 years due to follicle depletion or dysfunction.^1^ Oocyte depletion may be due to low initial numbers or accelerated loss. The function of the ovary may fluctuate in this state before failure, hence the recent preferred usage of the term POI (terminology used in this article will be consistent with references). POI with possible ovarian failure is a devastating diagnosis for a young woman’s health and hopes of motherhood. The condition is important to identify and its causes are important to investigate and research for the preservation of future well-being. The physical, psychological, reproductive, and social impact is significant and will be greater when the condition develops in very young women and adolescents. Life expectancy may be reduced because of skeletal and organ effects. This impact will increase where diagnosis is delayed or the condition and its causes inadequately treated. Causation is unknown in 74% to 90% of cases^2,3^ and the background age-specific incidence of idiopathic premature ovarian failure (POF) in early to mid-adolescence is so rare as to be also unknown, with the annual incidence reported as 10/100 000 person-years up to age 30 years.^4^ The development of idiopathic POI and POF in a series of young teenagers after receiving the quadrivalent human papillomavirus (HPV) vaccine therefore has no age-specific background rates for comparison.

Each quadrivalent HPV vaccine is a recombinant protein particulate vaccine, containing 20, 40, 40, 20 µg of the major capsid (L1) protein of HPV types 6, 11, 16, and 18 respectively, 225 µg aluminum hydroxyphosphate sulfate, 9.65 mg sodium chloride, 780 µg; l-histidine, 50 µg polysorbate 80, and 35 µg sodium borate (“Gardasil,” “HPV4,” “4vHPV”). It is recommended to young women for its protective role against the 2 most common HPV oncogenic types, HPV 16 and HPV 18. This vaccine could potentially prevent 70% of cervical cancer.^5^ Protection against 2 other HPV types (6 and 11) causing genital warts is included in HPV4. Since 30% of cervical cancer may still occur in vaccinated individuals, papanicolaou smears to the seventh decade are still required. Prior to vaccine introduction, the incidence and mortality rate of cervical cancer were steadily declining. These rates more than halved in the decade prior to 2000 in the 20- to 69-year age group in Australia and 578 new cases were diagnosed in 2000.^6^ The incidence was highest in remote areas,^7^ with the risk of death from cervical cancer for an Indigenous woman in Australia 6 times that of a non-Indigenous woman. In 1989, it was estimated that cervical cancer pap screening could potentially prevent 90% of squamous malignancies.^8^ Increasing success of the Australian National Cervical Screening Programme has been moving toward this capacity with prevention of 70% of squamous cell cancers in 1998,^9^ up from 46% in 1989. In 2002, the Australian incidence of cervical cancer was 6.2 per 100 000 women^8^ and the mortality rate 1.7 per 100 000 women. In 2011 in Australia, there were 229 deaths from cancer of the cervix.^10^ Five-year relative survival is 72.1%.^11^

A consideration of vaccine benefit versus vaccine risk requires high-quality safety evidence. This case series is therefore presented for its possible significance to young women’s health and fecundity. The limited capacity of existing HPV4 research to attest to ovarian safety together with factors that impede vaccine adverse event reporting could affect the quality of information supplied to informed consent. This second case series of adolescent POI/POF increases evidence suggesting that the hypothesis of an association between HPV vaccine and premature ovarian demise needs to be tested.

## Background

Early symptoms and signs of POI vary and a delay in presentation and diagnosis of POI is common. It has been observed that 92% of women with idiopathic POF describe an altered menstrual cycle as their initial symptom.^12^ A total of 58% have described amenorrhea lasting 3 months or longer as the presenting symptom and 29% have described oligomenorrhea as the presenting symptom. Polymenorrhea, infertility, metrorrhagia, and vasomotor symptoms were less common presentations. In all, 25% of karyotypically normal women with noniatrogenic POF took more than 5 years from onset of a menstrual cycle abnormality for the diagnosis to be established. The median duration to diagnosis was 2 years. Overall, 57% of women with POF required 3 or more clinician visits prior to laboratory testing and 61% of women reported seeing 3 or more clinicians prior to diagnosis. Noninvestigation of new menstrual pattern abnormalities in young women may be due to a low perception of importance by the patient or low perceived importance by the physician. It has been observed that 39% of women developing amenorrhea consult a doctor.^13^ Similarly, clinicians appreciate that some 4% of reproductive-aged women may miss 3 periods each year.^14,15^

Since the incidence of POF increases with age, we need finer gradations of incidence for very young teens at 13 and 14 years of age in whom this condition following HPV4 has been reported.^16,17^ The unknown prevalence of idiopathic premature ovarian failure in the early to mid-teenage HPV4 vaccine target group renders adverse event analysis methods such as “rapid cycle” vaccine event analysis inapplicable.^18^

Premature ovarian insufficiency has serious health implications. A Swedish study of 22 000 postmenopausal women suggests those entering menopause aged 40 to 45 years have a 40% increased risk of cardiac failure than those entering menopause at age 50 to 54 years.^19^ For every year delay in the onset of menopause the rate of cardiac failure was lowered 2%. The cardiac implications for teenagers entering menopause have yet to be defined. Altered ovulatory and menstrual patterns also lead to accelerated loss of bone density and increased wrist and hip fractures in later life.^20^ POF is one of the greatest risk factors for osteoporosis.^21^ Furthermore, lowered bone mineral density begins with diminished ovarian function before the onset of amenorrhea^22^ and suboptimal bone density in teens is a factor in the development of osteoporosis.^23^ Other health implications of POI will differ by cause.

Published case reports have considered a possible link between quadrivalent human papillomavirus vaccine and premature ovarian failure.^17,24^ Declining menstrual function in girls aged 14, 15, and 20 years followed HPV4 vaccination and preceded POF in the previous case series. The formerly published *BMJ* case report of a 16-year-old with irregular menses gradually progressing to oligomenorrhea, amenorrhea, and POF after HPV4 was the first such case presenting to this practitioner and is therefore summarized as “Case 1” below.

This case series presents 3 young women who consulted a primary care general practice in rural New South Wales, Australia. Two experienced a duration of cycle disruption progressing to amenorrhea and 1 had an unknown prodrome to amenorrhea due to oral contraceptive pill (OCP) usage. These symptoms followed HPV4 vaccination. The girls are not known to be related and reside 40 to 500 km apart (1 patient was holidaying). Vaccination batches that were identified were dissimilar and administered in locations 3, 500, and 570 km from this attending practitioner.

## Case 1

This case has previously been published in the *BMJ Case Reports*.^24^ It was the initial presenting case diagnosed in this series. Therapeutic Goods Administration of Australia adverse event report reference number is 285383.

Menarche at age 13 years in 2007 was followed by light periods, which became heavier and regularized over the next 12 months. HPV4 was administered in February, May, and August of 2008 (Department of Health New South Wales. 2011). Cycles became irregular early in 2009 and become scant and infrequent in 2010. Menstruation ceased in January 2011 and hot flushes commenced. There was no past history of significant illness or surgery. She was a nonsmoker, took no medications, and had no history of injury. Body mass index was 22.6 kg/m^2^. There was no family history of premature menopause. At her initial consultation for oligomenorrhea becoming amenorrhea, she was prescribed the OCP without investigation. She was not sexually active.

She declined the OCP and consulted a second clinician. Investigations revealed that follicle-stimulating hormone was 108 U/L (menopausal range 20-140 U/L); luteinizing hormone was 31 U/L (menopausal range 10-65 U/L); estradiol was low at 63 pmol/L (normal follicular range >110 pmol/L, menopausal range = 40-200 pmol/L). Progesterone was 1.1 nmol/L (menopausal range <2.2 nmol/L). Anti-Müllerian hormone was <1.0. There were no antiovarian antibodies or antiadrenal antibodies detected. Thyroid peroxidase antibodies were 2 IU/mL and thyroglobulin antibodies were 44 IU/mL (levels up to 100 IU/mL can occur in normal subjects). A pelvic ultrasound was reported normal. Full blood count, renal, liver and thyroid function, and prolactin were normal. Premature ovarian failure was diagnosed at age 16 years. Some irregular anovulatory pattern bleeds occurred before commencement of hormone replacement therapy.

Karyotype was 46XX. Galactosemia testing was negative. Fragile X testing was normal.

This girl was counseled about the need for bone strength preservation. Her bone mineral density testing suggested femoral neck to be in the low range for age, height, and weight at 0.766 g/cm^2^ and lumbar spine bone mineral density to be normal for height and weight but lower than the expected range for age at 0.903 g/cm^2^. She is considering ovarian tissue cryopreservation.

## Case 2

An 18-year-old young woman presented with 6 months amenorrhea. Menarche had occurred at age 11 years. She suffered from mild cerebral palsy (possibly due to low birth weight of 1.88 kg at 38 weeks’ gestation), mild asthma, Asperger’s syndrome, anxiety, and epileptic events from age 14 to 15 years, considered secondary to cerebral palsy. She had a ruptured appendix at age 12 years. There was no other significant past history; no drug usage; she did not smoke or drink alcohol, and had not become sexually active. Sertraline was used for treatment of anxiety from 2009 to 2010 followed by fluoxetine. There was a family history of osteoarthritis and osteopenia and of pancreatic cancer; no family history of premature menopause. The OCP was commenced at age 12 years. The attending gynecologist recorded “although her periods were reasonably normal, she was put on the pill (20 µg ethinyloestradiol and 100 µg levonorgestrol) the next year because coping with her periods made her anxiety and depression symptoms worse.” Her first HPV4 vaccination was administered at age 12 years and 9 months; the second vaccination near her 13th birthday, and the third vaccination at age 13 years and 5 months. The first HPV4 vaccination was given concomitantly with hepatitis B vaccination in the other arm. OCP usage continued for 2 years to age 14 years. It was briefly ceased at age 14 and this was followed by 3 months amenorrhea. OCP was then resumed without further investigation. At age 18 years, the OCP was again ceased and amenorrhea again ensued.

Follicle-stimulating hormone 1 month later was elevated at 44.5 IU/L (menopausal range is 25-130 IU/L). Luteinizing hormone was 29.2 IU/L (basal range 2.0-12, midcycle peak range 8.0-90, postmenopausal range 5.0-62 IU/L). Estradiol was 157 pmol/L. She continued amenorrheic and presented again for investigation 6 months later. At this time, follicle-stimulating hormone remained elevated at 34 IU/L. Luteinizing hormone was elevated at 46 IU/L. Estrodiol (Oest2) was 413 pmol/L and progesterone 2 nmol/L. Anti-Müllerian hormone was 1.5 pmol/L (14.0-30.0 pmol/L normal; levels <14 pmol/L suggest diminished ovulatory reserve) tested by Beckman Coulter Gen II ELISA assay. Anti-Müllerian hormone repeated 6 months later was <1 pmol/L and estradiol was <37 pmol/L. At this time and at age 18 years POI was diagnosed.

Full blood count, iron levels, liver function, blood glucose, and renal function were normal. Thyroid-stimulating hormone was normal 0.4 mIU/L (normal 0.3-3.5 mIU/L) and thyroid antibodies were normal. Prolactin was normal 294 mIU/L. Testosterone 0.9 nmol/L (normal 0.2-1.8 nmol/L); free androgen index 2.6 (normal 0.3-4.0); iron studies were normal. There were no antiadrenal or antiovarian antibodies detected. Morning cortisol was 218 nmol/L (normal range 160-650) nmol/L, ACTH 14 ng/L (normal range 9-51 ng/L), growth factor-1 27 nmol/L (normal range 21-76 nmol/L). Pelvic ultrasound performed at the time when the anti-Müllerian hormone level was 1.5 showed a normal uterus with an endometrial echo of 8.2 mm. Transvaginal ultrasound was declined and the left ovary was not visualized. The right ovary was 3.1 cm^3^ in size and there was a 9-mm follicle within it. Brief menstrual bleeds then occurred for 4 months before amenorrhea resumed.

Testing for Fragile X revealed 2 normal-sized triplet alleles 23 and 37 cytosine–guanine–guanine n repeats (the normal zone is <44). Testing for galactosemia showed a normal Gal-1-P uridyl transferase-RC at 0.31 U/g hemoglobin (normal range 0.26-0.52 U/g). Records reported a vitamin B_12_ deficiency at age 16 years, but levels of vitamin B_12_ were within normal limits at 275 pmol/L (normal range 135-650 pmol/L). Karyotype was established as 46XX.

This young woman elected to undergo right ovary cryopreservation through Monash IVF in the hope that future developments, such as stimulation of ovarian stem cells, may be of later benefit. She was not deemed a suitable candidate for gonadotropin stimulation for oocyte preservation due to the undetectable anti-Müllerian hormone level. The pathologist described the macroscopic appearance of the ovary as “cystic and disrupted.” Microscopic histology of three right ovarian biopsies reported fibrovascular connective tissue with no primordial follicles in the ovarian cortex of sample one. Ovarian sample 2 reported a cystic follicle and a cystic corpus luteum but no primordial follicles within surrounding parenchyma. Ovarian sample 3 reported “fibrofatty connective tissue only. No ovarian parenchyma is identified.” Summary: “Levels through all tissue containing ovarian parenchyma show a single primordial follicle. No other follicular structures are identified.” No samples contained evidence of atypia or malignancy (Sullivan Nicolaides Pathology. Brisbane, Queensland, Australia). Personal communication with the reporting pathologist confirmed no lymphoid or granulomatous inflammation and suggested the ovarian appearance was “consistent with that of a woman in her late forties.”

She has been counseled about bone density preservation and the need for hormone replacement therapy. This case was notified to the Therapeutic Goods Administration (TGA) of Australia in January 2014 (reference number 333136) as diagnosed POI. Its listing as “amenorrhoea” on the TGA database in May 2014 was later altered to POF (July 2014).

## Case 3

Menarche had commenced at age 10 years, and was followed by regular menses. The first 2 HPV4 vaccinations were received at age 14 years and the third vaccine after turning 15 years in 2008 (Department of Health New South Wales. School vaccination programme. Vaccinations administered February 18, May 23, and October 24 in 2008). The patient reports “prior to this, my periods were like clockwork.” The period due after the third vaccination dose was 2 weeks late and was the first late period she had experienced. The next period occurred 2 months later. The next and final menstruation occurred 9 months later, approximately 1 year after completion of the third HPV4 vaccination. Hot flushes developed and 10 kg weight gain was noted over the next year. Previously present acne improved. Pelvic ultrasound was unremarkable apart from a 3.7-mm endometrial width and the absence of visible ovarian follicles. She had not become sexually active, had no history of drug or alcohol usage and there was no history of trauma, surgery or of significant past illness. There was no family history of premature menopause. She was allergic to benzoyl peroxide. POF was diagnosed just before her 17th birthday.

At age 15 years, initial testing was undertaken: testosterone was 1.1 nmol/L (normal range <2.6), sex hormone binding globulin 41 nmol/L (normal range 20-118 nmol/L), free androgen index 2.7% (normal range <7.2%). There is no significant further testing until nearly 17 years of age: prolactin 160 mIU/L (normal range 40-570 mIU/L), thyroid-stimulating hormone 1.1 mIU/L (normal range 0.5-4.5 mIU/L), dihydroepiandrostenedione (DHEA-S) 3.5 µmol/L (normal range 3.6-9.8 µmol/L), androstenedione 1.8 nmol/L (normal range 1.0-11.5 nmol/L), testosterone <0.7 nmol/L (normal range <3.2 nmol/L), serum hormone binding globulin 32 nmol/L (normal range 30-90 nmol/L), free androgen index <2.2%, luteinizing hormone 32.8 IU/L (midcycle range 17.7-47.5; postmenopausal range >9.3 IU/L), follicle-stimulating hormone 73.8 IU/L (midcycle range 9.6-24.1; postmenopausal >50 IU/L), estradiol <100 pmol/L (midcycle range 500-1500; postmenopausal <100 pmol/L). Estradiol (radioimmune assay) <10 pmol/L. Repeated hormone levels 7 weeks later revealed luteinizing hormone 42.9 IU/L, follicle-stimulating hormone 61.8 IU/L, and estradiol 18 pmol/L. Antiovarian antibodies were negative <1:10 and antiadrenal antibodies were negative. Anti-Müllerian hormone level was unrecordable. Bone mineral density scan was reported normal at age 17 years and 1 month (*z*-score 0.9 for lumbar spine and 1.4 for “whole body”).

When reviewed in the Department of Clinical Endocrinology at Westmead Hospital, New South Wales, it was determined that she would not respond to gonadotropin stimulation for oocyte collection for cryopreservation. She has been counseled about the need for bone preservation and is currently on hormone replacement therapy. This case was reported to the Therapeutic Goods Administration of Australia in April 2014. No response was received and the case was renotified to the TGA in June 2014 and to the New South Wales Chief Medical Officer. Reference number and notification response are awaited. Consultation for ovarian cryopreservation has commenced.

## Discussion

Consideration of the possible significance of this second case series of idiopathic POI/POF after HPV4 requires review of preclinical and clinical safety studies identified at licensing^25^ and review of larger postlicensing safety studies. A summarized report of existing HPV4 research in relation to the very young ovary was presented by this author at the 18th World Congress of Controversies in Obstetrics, Gynecology and Infertility in October 2013^26^ and to the Brighton Collaboration Journal Club (as author response to review of *BMJ* September 2012 Case Report).^27^

### Preclinical Studies

Safety assessment of a new vaccine begins with preclinical studies for toxic effects in rodents. After diagnosis of case 1, and in response to a query from this patient, rodent ovarian histology after HPV4 vaccination testing was sought. No histology report of the vaccine-tested rodent ovary was available under Freedom of Information Request to the Therapeutic Goods Administration of Australia.^28^ There is no cellular observation available on tested rodents’ ovaries beyond a numbering of corpora lutea present on the ovary at caesarian section.^29^ Five-week-old tested rats conceived only 1 litter before euthanasia.

It is unfortunate that available toxicology studies only provide histology of the male rodent reproductive system after HPV4 vaccine^30,31^ and not of the female rodent reproductive tract or ovaries. Vaccine-tested rat ovary histology reports would have been useful to consult to better understand any possible link between cases of teenage premature ovarian insufficiency and rat vaccine effects.

Published Sprague-Dawley rat testing for HPV4 vaccine fertility safety comprised 2 control groups and 2 vaccine groups.^32^ Control group 1 was given a formulation of phosphate-buffered saline as placebo (the chemical formulation selected is not stated). Control group 2 consisted of the carrier solution components of Gardasil. It contained “aluminum (0.45 mg per mL), sodium chloride (18.7 mg/mL), sodium borate (70 mg/mL [sic]), l-histidine (1.55 mg/mL), and polysorbate 80 (100 µg/mL).” Vaccine group 1 consisted of rats only given the vaccine after their first mating and resultant conception. Vaccine group 2 rats received 2 vaccine doses 5 and 2 weeks prior to first mating/conception and at 6 days after conception and on lactation day 7.

Twenty-two rats within each of these 4 groups were assigned to caesarian section, and 22 from each group were assigned to give live birth before postweaning euthanasia. In the caesarian section data,^32,33^ the total number of corpora lutea present in the group of 22 rats not vaccinated before mating, of whom all 22 fell pregnant at mating, was 366. The total number of corpora lutea present in 22 rats who received the first and second vaccinations before mating, of whom 20 fell pregnant at mating, was 326. The ratio of corpora lutea per rat that did fall pregnant was 16.30 (±2.5 SD) for those receiving 2 vaccinations before mating, and for those not vaccinated prior to mating was 16.63 (±2.3 SD). While these were only small differences of corpora lutea numbers, it is not known whether administration of the complete 3-dose vaccination course to test fertility may have shown a more significant disparity. The overall fecundity index of rats who received two thirds of the vaccination course prior to mating was 95%, the lowest of the 4 groups and very slightly lower than the fecundity index of 98% in rats who received no vaccination prior to mating. In controls 1 and 2, the fecundity index was 97% and 98%, respectively. In preclinical fertility studies submitted at licensing, no rats were tested with the complete vaccination course, with representative interval administration, prior to mating. The study concludes that vaccine rodent fertility testing conferred “a safety margin of 200-fold by body weight for adolescents.” “Guidance for Industry” research guidelines state “where possible we recommend that you administer the maximum human dose (eg, 1 human dose = 1 rabbit dose) regardless of body weight.”^34^ The reason for omission of the third vaccination dose prior to measuring the rats’ capacity to conceive is unclear.

The 200-fold safety prediction was derived from the 0.25 kg weight of a rat compared with the “average body weight of an adolescent girl (50 kg).” The HPV4 target girl group is aged from 9 years and administration in Australia is to girls aged 12 and 13 years under the National Immunization Programme. The 50th centile weight of 9-year-olds is 28 kg, of 12-year-olds is 42 kg, and of 13-year-olds is 46 kg.^35^ Australian age-specific weights therefore also reduce modeled calculations of fertility safety.

Long-term fecundity studies of vaccinated female rodents’ duration of reproductive lifespan, recorded numbers of litters and pup numbers in subsequent litters was also requested under the original freedom of information application but were unavailable.

### Clinical Studies

Research consideration of ongoing female fertility was similarly absent from phases II and III clinical safety studies. The capacity of safety studies to assess ovarian function, particularly of the target age group, was reduced by several factors. The phase II and phase III studies identified as safety studies at the time of licensing^25^ by the Vaccine and Related Biological Products Advisory Committee (VRBPAC) to the Food and Drug Administration are study protocols V501 007,^36^ 016,^37^ 018,^38^ and 013,^39^ and 015,^5^ respectively. Only protocols 016 and 018 studied safety in the young female vaccine target group. Mean ages in these groups were 12.6 and 11.9 years, respectively. It is not clear what proportion of these were postmenarche.

In protocol 016, 240 girls (aged 10-15 years) were left in the study at 12 months, comprising 47.4% of screened healthy participant younger girls. Immune response data were collected through month 7, and safety data through to month 12. More than 52% were lost from the 12-month safety follow-up instituted as a protocol amendment. Loss of the majority of participants to safety observation significantly compromised this trial as a safety study of younger adolescents forming the vaccine’s target group. One girl in this study experienced vaginal hemorrhages meeting Serious Adverse Event Criteria^40^ after second and third vaccinations. These were initially deemed vaccine related, but subsequently considered by gynecological review^37^ to have been related to a preexisting condition not excluded at general health screening. Protocol 018 fully vaccinated 587 girls. A total of 52.3% of enrolled girls were aged 9 to 12 years. It is not clear what proportions of girls in these target group safety studies could potentially have reported menstrual cycle patterns or aberrations of patterns. Similarly, health interviews with the participants 18 months after the first vaccination may not have been able to determine menstrual abnormalities while cycles are commencing or establishing ovulatory patterns.

Given the masking effects of hormonal contraception on ovarian function it is relevant that contraceptive hormone usage was reported at 58% to 60% of vaccine recipients in safety trials at baseline interview in phase III studies.^41^ This rose to 68% to 83% of participants in the 2 substudies of protocol 013.^42(p143)^ In all, 75% to 82% used hormonal contraception within 15 days of any vaccination in trial 007,^42(p216)^ and more than two thirds recorded concomitant hormonal contraception usage within 14 days of any vaccination in protocol 015.^42(p244)^ Phase III studies’ participants, mostly 16 years and older, were required to use effective contraception for at least 7 months. A major review of the HPV4 vaccine safety profile reports: “new medical conditions were not considered adverse events if they occurred post month 7, or were not considered by the investigator to be vaccine related.”^43^

The design of safety studies with use of a vaccine report card further restricted the recording and reporting of menstrual dysfunction. The largest safety study, phase III study protocol 015, enrolled older females predominantly aged 16 to 23 years (1 was 15 years old, 46 were older than 23 years) of whom 5916 completed the 3-dose HPV4 vaccination period and 5953 completed placebo dosages.^42(p58)^ A subgroup selected from among these formed the Detailed Safety Cohort. It followed 448 recipients of at least 1 vaccination and 447 control recipients, asking them to record nonserious adverse events (NSAEs) for 2 weeks after each vaccination on a vaccine report card. Serious adverse events (SAEs) in the 2 weeks following each vaccination were also recorded.^5^ Participants not included in the NSAE substudy were solicited only for SAEs occurring within 2 weeks after each vaccination.^42(p52)^ All SAEs that were considered to be potentially related to administration of the vaccine were to be reported throughout the study. However, menstrual cycle disruption, oligomenorrhea, and amenorrhea will not signal as SAEs by definition. SAEs are defined as life-threatening, resulting in death, permanent disability, congenital anomaly, hospitalization, prolongation of hospitalization, or necessitating medical or surgical intervention to prevent one of these outcomes.^44^ The use of a vaccine report card to record other adverse events occurring within 2 weeks of each vaccination has limited ability to detect diminishing menstrual cycles. This is a weakness in the safety design of clinical trials. It would not have detected the menstrual cycle decline evident in the cases of premature ovarian insufficiency presented in this series. Protocol 018 VRC prompted for additional information such as headaches, rashes, muscle/joint pain, and diarrhea that occurred within 14 days but not menstrual aberration.

When the Center for Biologics Evaluation and Research requested an analysis of autoimmune conditions over the entire safety database, the sponsor noted that “there were subjects with additional new medical conditions that were not reported in the Clinical Study Reports for 011 and 012 [within protocol 013]. These included two subjects with amenorrhea.”^42(p198)^

Longer term follow-up beyond the vaccination interval was limited to SAEs. Protocol 015 mean follow-up was 3 years from first vaccination for SAEs. The second largest study, protocol 013, fully vaccinated 2582 women^42(p136)^ and vaccine report cards recorded NSAEs for 2 weeks after each injection. Lack of long-term follow-up is identified as a limitation of this study.^39,42(p136)^

Underrepresentation of the vaccine’s target age group, incomplete and short-term follow-up, definitional limitations, hormone usage, fortnight restrictions of vaccine report card documentation and the decision not to report new medical conditions as adverse events which occurred post month seven from first vaccination compromised safety studies’ observation of ovarian health.

### Vaccine Components Used as Safety Study Controls

The choice of placebo affects the validity and quality of scientific information available from placebo-controlled studies. The control in any experiment should lack the factor being tested. The placebo that formed the control selected for phase III safety studies of Gardasil (older girls) was the aluminum adjuvant present in the vaccine solution, amorphous aluminum hydroxyphosphate sulfate. The selection of aluminum as a control in vaccine studies is at variance with the scientific principles of a control. The placebo in the only controlled study of very young girls was the remainder of the vaccine carrier solution: “The placebo used in this study contained identical components to those in the vaccine, with the exception of HPV L1 VLPs and aluminum adjuvant.”^38^ It contained 50 µg polysorbate 80 (polyoxyethylene sorbitan mono-oleate also known as Tween 80), 35 µg borax, 9.56 mg sodium chloride, and 0.78 mg l-histidine.

Safety studies identified at licensing did not compare HPV4 with normal saline controls. The second placebo contained several substances together with saline. The researchers’ reference to the “carrier solution” placebo conflicts with the licensing review. The Center for Biologics Evaluation and Research states, “Protocol 018 provides saline placebo-controlled safety data for subjects 9 to 15 years. This is of particular interest because the other studies used alum placebo as a safety comparison.”^42(p330)^ Subsequent reviews of safety studies also claim a saline placebo was the comparator of younger girl safety studies and variously refer to this placebo control as “non-aluminum containing (saline) placebo”^43^ and “saline placebo.”^45^ Gardasil Product Information itself refers to the control as a “saline placebo.”^46^ Published safety studies only compared HPV4 vaccine with its own components. This may be significant since injected substances in both placebo control arms have either a suggested association with autoimmune ovarian damage^17^ or known direct ovarian toxicity.^47^ Completed vaccine and placebo courses each administered 675 µg of aluminum to older girl safety study participants; or components including 150 µg polysorbate 80 to all 9- to 15-year-old safety study participants.

When polysorbate 80 (“Tween 80”) was injected into newborn rats, it caused similar ovarian damage to injected diethylstilboestrol. Rat ovary effects occurred at all doses tested over a tenfold range.^47^ Since this study provides a relevant ovary histology report of a substance in HPV4 it bears detailed consideration. 1%, 5%, or 10% solutions of polysorbate 80 at 0.1 mL per rat were injected into rats at 4, 5, 6, and 7 days after birth. The oestrous cycle was examined at weeks 10, 14, and 18 of age. Findings were compared with control rats given no treatment; negative controls given water injections; and a “positive” control group given a formulation of 50 µg diethylstilboestrol. Rats injected with polysorbate 80 had an oestrous cycle ranging from 9 to 14 days, compared with 4.3 days average length throughout the test in untreated controls and 9.4 days in diethylstilboestrol injected rats. Postmortem conducted at 20 weeks of age on “Tween”/polysorbate 80 tested rats reported

1. All Tween-treated groups showed a statistically significant (*P* < .001) decrease in the relative weight of the ovaries in comparison with the untreated control. The relative weight (% of body weight) was slightly lower in the 1% Tween 80–treated groups than in the 10% Tween 80–treated groups.
2. In the group of 6 rats given the lowest dose of Tween 80 “in two rats the uterus was enlarged and had a marked vascular pattern.”
3. The 5 rats given diethylstilboestrol showed “microscopically degenerated follicles in the ovaries with complete absence of corpora lutea. Findings in the ovaries similar to those in the positive control [diethylstilbestrol control] group were also observed in all of the groups given Tween 80.”
4. Abnormal histological findings in the cells lining the uterus were observed in all 17 rats given Tween 80 and resembled the abnormal histology observed in diethylstilboestrol-treated rats, which had high cylindrical epithelial cells and some mitoses. The study concluded, “4-day administration of Tween 80 to female rats during the period crucial for the development and function of reproductive organs accelerates the maturation of these organs.” As well as a prolonged oestrous cycle researchers also noted induction of persistent vaginal oestrous. This was slightly more marked in the 1% solution of Tween 80 than in the 5% or 10% solutions. Statistically significant increased weight of the adrenals (*P* ≤ .05) was also noted in the 1% polysorbate injected rats.

No dose response curve was identified. This chemical is present in orally ingested medicines and foods, but did not affect rat reproduction when subject to digestive processes at up to 5% of their oral intake. It did decrease rat reproduction at 20% of their oral intake.^48^ It is not known whether some protection may be conferred by digestive processes to smaller loads of polysorbate 80 that is not present in the parenteral route of administration to young female rats. This research highlights 4 issues. First, the scientific role of control groups and placebo selection. Second, a possible confounding effect of polysorbate 80 used as placebo in younger girls’ HPV4 safety trials. A potential ovarian toxin in both control and vaccine arms could obscure the already limited ability to observe risk differences of adverse menstrual events. Third, the absence of crucial histological reporting of the rodent ovary after HPV4 vaccination containing 150 µg polysorbate 80. Fourth, whether clinical investigators of vaccine safety considered the potential ovarian effects of this vaccine component when determining a “likelihood” relationship between menstrual aberration and the study vaccine. Safety trial investigators determined the relationship of both Vaccine report card documented adverse events and new medical conditions before month 7 to vaccination, based on criteria of “likely cause,” exposure, time course, and rechallenge. Licensing bodies asserting “no biological plausibility”^49,50^ of ovary effects arising from vaccine constituents accept a null hypothesis at odds with existing research. This may also reflect research investigator considerations of “likelihood.”

Histologically evident toxic ovarian effects of polysorbate 80 evidenced 5 months after serial injections into very young rats have not been compared with the histological effect of the HPV4 vaccine course containing 150-µg dosage. The relevance of polysorbate 80 ovarian damage to the cases presented here is unresearched and unknown and assurances of “no biologically plausible” link between HPV4 vaccine and ovarian effects cannot be given.

The role of the aluminum adjuvant as a safety study placebo also requires consideration. The development of an “autoimmune/inflammatory syndrome induced by adjuvants” (ASIA) has been postulated by some immunologists to be implicated in the development of premature ovarian failure.^17^ The 3 young women in the previous published case series of POF following HPV4 had associated symptomatology which fulfilled criteria for this syndrome. These criteria include clinical signs (such as neurological, sleep, or cognitive disturbances, myalgia, arthralgia, fatigue, and fever) with a major feature of prior exposure to external stimuli such as infection, vaccine or vaccine adjuvants, and possible other autoimmune phenomena.^51^ Existence of this syndrome is under dispute, but proponents suggest autoimmunity may be induced in this context in genetically predisposed individuals. Antiovarian antibodies were found in the 15-year-old girl diagnosed with POF in that series. A possible autoimmune implication of injected aluminum reinforces the scientific principle that placebos should not contain the factor being tested. Respect owed to this principle is further endorsed by findings that “the structure of the ovary was disrupted” in rats exposed to subchronic ingestion of aluminum chloride.^52^ Other associations with this aluminum salt included reduced levels of alkaline phosphatase, acid phosphates, and ATPase and lowered protein expression of follicle-stimulating hormone receptor and luteinizing hormone receptor. The selection of aluminum as the safety comparator may have confused safety trial outcomes. Its use as a placebo is therefore questionable.

### Postlicensing studies

Two large safety studies, sentinel cohort follow-up, reviews of existing research, and vaccine adverse event reporting systems have reported on postmarketing vaccine safety.

The first of the 2 largest post marketing studies sought to evaluate vaccine safety “during the course of routine clinical care” by reviewing presentations at emergency departments and hospitalizations from within a cohort of 189 629 vaccinees.^53^ This group included 44 000 females who had completed 3 vaccinations. Eleven- to 12-year-olds comprised 4.3% of the total vaccine group. Emergency department visits are not the consultation context for seeking medical management of altered menstrual cycles, oligomenorrhea, or amenorrhea. These conditions rarely require hospitalization. This study had no capacity to evaluate ongoing ovarian health or safety. Further analysis of these emergency department presentations/hospitaliza-tions to review the risk of 16 autoimmune conditions did not include ovarian dysfunction or failure.

The largest and most recent published cohort study from Denmark and Sweden of 997 585 girls measured incident hospital diagnosed autoimmune, neurological, and thromboembolic events.^54^ Menstrual cycle aberrations indicative of ovarian malfunction were not included and, again, do not usually present to emergency and hospital settings. This study of approximately 1 million girls sheds no light on reproductive function or egg-bearing capacity.

The sentinel study of 577 girls from protocol V501 018 was to provide the first long-term data of vaccinated adolescents. It assessed safety by monitoring for serious adverse experiences and pregnancy outcomes.^45,55^ The Nordic extension of the long-term follow-up of protocol V501 015^45^ addressed the hypothesis that Gardasil will remain effective for 14 years after vaccination. This long-term follow-up study will connect with National Hospital Registers in participating countries and cancer registries searching for adverse events such as deaths, hospitalizations, cancers and other safety outcomes. It has the capacity to search health-related registries to find “safety events of interest,” comparing adverse event rates to those in the age-matched general population. Ovarian function is not recorded in its research focus. Furthermore, data search of ovarian insufficiency if undertaken may be impeded by evidenced delays to diagnosis within 5 years,^12^ OCP usage and lack of incidence statistics in an age-matched population.

Postlicensure monitoring is relied on to detect rarer adverse events. The Vaccine Safety Datalink^56^ has reviewed associations between HPV4 vaccination and outcomes prespecified as Guillan-Barre, stroke, venous thromboembolism, appendicitis, seizures, syncope, allergic reactions, and anaphylaxis. Ovarian dysfunction was not studied and rare events need background incidence rates for comparison. Monitored outcomes were those with relatively acute onset, which could “represent a biologically plausible association with vaccination.” Rapid cycle analysis of vaccine safety datalink information requires comparison between vaccinated and unvaccinated groups.^57^ The Clinical Immunization Safety Assessment Network reviews SAEs reported to the Vaccine Adverse Event Reporting System (VAERS) following immunization and have therefore reported on deaths, venous thromboembolism, neurological, and allergic outcomes.

The VAERS^58^ accessed August 2013 noted 104 cases of amenorrhea following HPV4. Less than 9% had a reported return of menses. Only 1 girl out of 105 amenorrhea notifications had a follicle-stimulating hormone level recorded. This was “elevated at 72” (no units given). No notifications had an anti-Müllerian hormone level reported. A comparison of adverse events following immunization reported to VAERS with adverse events following immunization reported to the World Health Organization “Vigibase” reveals similar proportions of notifications.^59^ As with all passive reporting systems, reliance is placed on voluntary reporting accessing the reporting process. These reports often derive from a population of unknown size. It is not possible, therefore, to determine the incidence of these events or to assess causality. The chief function of adverse event reports and of case reporting is to generate hypotheses for further study.

## Considerations

The great quantity of research concerning HPV4 vaccine does not necessarily establish a comprehensive, qualitative safety assurance. The administration of vaccines to all well prepubertal and peripubertal young persons necessitates a consideration of reproductive health that has not been met in the context of ovarian health. Selection of large numbers of participants may not produce the best data if the vaccine target group is underrepresented, or if research for adverse events is focused on hospitalizations and emergency settings rather than routine primary care settings—the context for many disease category presentations. Regardless of vast data, pre- and postlicensing studies have not assessed ovarian safety. Neither vaccine target age group study considered the proportion of girls postmenarche. Research reviews have not always analyzed safety study design quality. An Australian review of this vaccine’s safety research spoke of “impressive clinical trial results” conducted before licensing.^60^ However, younger person safety studies comprised 2 phase II studies of which only 1 had a “control” group, with very small numbers of young females receiving all 3 vaccinations. One of these 2 studies had lost more than half to 12 month follow-up even though these 10- to 15-year-olds were to be followed for 1 year after first vaccination for safety related data.^37^ Only 40.4% of boys studied, 205 in total, completed 12-month safety follow-up in this designated safety study in which one 15-year-old boy died suddenly 27 days after his second vaccination.^37^ With no clinical findings at autopsy, the investigator determined his death was unrelated to the study vaccine “because of the lack of any plausible or temporal relationship.”

Other considerations await research. The relevance of claimed detection of HPV L1 gene DNA sequences in Gardasil vials in separate studies is unknown.^61,62^ A recent report states “preliminary data showed the presence of contaminating HPV L1 DNA in all tested different batches of Gardasil® Vaccine from France. Our observations confirm independently and extend the previous observations by Lee SH.” Researchers concluded “the persistence in muscle tissue of residual HPV DNA fragments is uncertain after intramuscular injection and requires further investigation for vaccine safety.”

The occurrence of increased adverse vaccine events in girls who had not previously been exposed to the HPV vaccine viral types before vaccination also has unknown relevance. Food and Drug Administration summary of safety trials reported most adverse events occurring in girls naïve to the injected vaccine HPV types prior to vaccination.^42(p288, p432)^ Those who were polymerase chain reaction (PCR) negative and seronegative to all 4 vaccine HPV types at baseline reported the highest incidence of systemic adverse events, the highest proportion of “moderate to severe” systemic adverse events and the highest incidence of headache after Gardasil when compared with groups who evidenced prior HPV type exposure at baseline. The difference in adverse event recording between those naïve to the HPV 6, 11, 16, 18 types and those who had been previously exposed was most marked in the “Detailed Safety Cohort” of protocol 015 (United States).^42(p93)^ In all, 63.8% of previously unexposed (seronegative/PCR negative) vaccine recipients recorded clinical adverse events after any injection. This compared with 51.5% of previously exposed (seropositive or PCR positive) recipients. The disparity within control groups was less marked. The Detailed Safety Cohort analysis records adverse clinical events in 60.4% of HPV naïve recipients of the aluminum control compared with 56.1% of HPV-exposed recipients. In the vaccine cohort, the disparity between the rates of clinical adverse events recorded in those who were naïve to HPV types 6, 11, 16, 18 prior to vaccination and those who were not naïve increased with each successive dose. The greatest difference was observed after dose 3, when 27.9% of naïve recipients had clinical adverse events recorded, compared with 16.8% of those who evidenced previous exposure to HPV types 6, 11, 16 and 18. Where only one HPV type was present in the tested vaccine, phase II study 005 of HPV 16 L1 VLP, there were no increased clinical adverse events in the sexually naïve. None of the 3 girls in the case series discussed was sexually active. The relevance of this status is not known. Since the preferred target group of the HPV4 vaccination program is virgins, and this group is less represented in safety studies, further clarification is appropriate.

These cases presented to a part-time general practitioner in a 5-doctor general practice. Cases 1 and 2 lived in different towns 40 km apart. Case 3 had been diagnosed elsewhere but her case had not been notified to the TGA. Her presentation, while holidaying, was stimulated by her awareness of case 1 in medical literature and by identification of the township of the author. The third case had passed unnoticed in the context of preceding HPV vaccination. The number of girls in the population who may have a similar unnotified diagnosis is not knowable. The pattern of ovarian demise here is not clear, but a gradual process is apparent. Lack of diagnosis in cases 1 and 2 prompted investigation in preference to further issuing of oral contraceptive prescriptions. OCP prescribing would delay appropriate diagnosis and management as well as notification of a possible adverse event. Therapeutic management was commenced with a more appropriate level of hormone replacement, attention to calcium, vitamin D, exercise, bone mineral density, and subsequent monitoring for autoimmune conditions that may be associated. Psychological health will also be monitored given the physiological and emotional effects of this diagnosis. Depressive symptoms were not found in these patients. Anxiety symptoms have been found in premature ovarian failure and psychosocial stress has scored higher during the year before cessation of menses.^63^

Diagnosis of idiopathic POI in mid-adolescence raises questions around future childbearing. Because of unrecordable anti-Müllerian hormone levels, 2 of these cases were not considered suitable for oocyte collection and cryopreservation. Recent studies of mouse oogonial stem cells have suggested the possibility of in vitro propagation and of future egg generation in vivo. Ovary tissue cryopreservation is being considered for future assistance in fertility preservation.^64^

## Future Research

Further research would consider a delayed onset of ovarian decline as suggested in this case series. The starting point of these girls’ anti-Müllerian hormone levels is not known, but the decline in case 2 from 1.5 to <1 in 6 months may reflect the gradual decline that has possibly taken 5 years to complete. Anti-Müllerian hormone levels are a biomarker of ovarian reserve, with 1 study suggesting peak levels at 15.8 years and a decline commencing after age 25 years.^65^ Their measurement may have a role to play in researching and monitoring ovarian demise and toxicity, since anti-Müllerian hormone levels strongly correlate with the existing antral follicle count and are therefore a quantitative measure of ovarian reserve.^66^ Rodent ovarian histology—similar to the manufacturer’s rodent testis histology report postvaccination—is also required. The delayed onset evidenced by this case series could also inform belated rodent ovary and fecundity studies to observe rodent ovaries and reproductive capacity at intervals after completed vaccination through their reproductive life span. Rodent fertility studies did not evaluate the standard vaccine course prior to conception, or the cumulative effect over time of 3 serial vaccinations, or the possibility of a delayed effect on reproductive capacity. Further research is needed to determine whether fecundity and fertility indices in rats are affected by the completed dosages administered to young teens as per industry guidelines.

Cohort studies of menstrual patterns in vaccinated and unvaccinated individuals are also required with timed anti-Müllerian hormone sampling. All such research should be wholly independent of commercial interests and manufacturer.

## Conclusion

It is not known whether idiopathic POI developing progressively in young teens following HPV4 is related to this vaccination. Case reports do not and cannot establish causation. It is known that safety research before and after licensing has inadequate capacity to determine ovarian safety. Small numbers of young persons represented in research, hormonal usage in older females’ studies, vaccine report card limitations, omission of a true placebo, inconsistent rodent toxicity studies, limitations of SAEs recording, subjective investigator decisions of likelihood and failure to record new conditions arising after month 7 as vaccine-related have weakened safety research. Diagnosis of premature ovarian insufficiency and failure is delayed in the general population and notified teenage amenorrhea is similarly underinvestigated in VAERS documentation. This primary care issue reduces the effectiveness of postmarketing surveillance. POF/POI notifications would be further compromised by OCP treatment of uninvestigated amenorrhea and hormonal contraception levels in the general population. Analysis of adverse event reporting is impeded by lack of background age-specific teen incidence figures. Long-term follow-up data after HPV vaccination has not surveyed ovarian function, recorded, measured, or analyzed symptoms or signs of dysfunction. Disparagement of adverse event reporting by licensing bodies’ instruction to health providers that “there is no biologically plausible way in which HPV vaccine could cause infertility” lacks science and compromises safety monitoring by undermining “reporting efficiency”^67^ safety signaling and informed consent. Public reassurance that “studies have not found ovarian failure to be associated with HPV vaccination”^68^ in the absence of sound research may be harmful to vaccine confidence. Edward Jenner, considered the father of vaccines, was known to say “let’s not speculate, let’s do the experiment.” Further studies are required to make any claims of ovary complications. Principles of informed consent, population health, and vaccine confidence require careful, rigorous and independent research to establish ovarian safety following HPV vaccination.
